# A review of nutrition labeling and food choice in the United States

**DOI:** 10.1002/osp4.374

**Published:** 2019-11-14

**Authors:** Alice Dumoitier, Vincent Abbo, Zachary T. Neuhofer, Brandon R. McFadden

**Affiliations:** ^1^ Carlsberg Groupe Lyon France; ^2^ Michel & Augustin Paris France; ^3^ Agricultural Economics Department Purdue University West Lafayette Indiana; ^4^ Department of Applied Economics and Statistics University of Delaware Newark Delaware

**Keywords:** nutrition, obesity, weight

## Abstract

A proliferation of processed food and labeling claims motivated the Nutrition Labeling and Education Act of 1990, which mandated the Nutrition Facts Label. Providing nutrition information is often put forth as a way to change food choice; however, despite efforts to provide dietary information using nutrition labeling, more than a third of the US has obesity and portions of the population continue to under consume vital nutrients. There has been progress beyond the Nutrition Facts Label in recent years with front‐of‐package labeling and menu labeling, which is crucial given changes in consumption trends for food‐away‐from‐home. Additionally, changes were recently made to the Nutrition Facts Label due to lack of awareness, understanding, and ability to effectively improve diet quality. This paper explores the literature to track the evolution of knowledge about attention to nutrition information and how nutrition information affects dietary choices.

## 1 INTRODUCTION

Despite rates of food insecurity decreasing from a recent high of approximately 15% in 2008 to approximately 11% in 2017,^1^ more than triple the rate (35%) of persons in the United States have obesity.^2^ The rate of persons with obesity indicates that many Americans are consuming enough calories to meet, or exceed, energy requirements. However, consuming an energy‐dense diet is not tantamount to consuming a nutrient‐dense diet.

The 2015 Dietary Guidelines Advisory Committee report determined “nutrients of public health concern” in the US and concluded the population underconsumes calcium, fiber, iron, potassium, and vitamin D while overconsuming saturated fat and sodium.^3^ The costs associated with malnutrition are not trivial or completely internalized by the malnourished. For example, the medical costs associated with persons with obesity every year is estimated to be $209.7 billion.^4^ Improved nutrition can decrease health care costs, for example, reducing sodium intake to the recommended daily value would save an estimated $18 billion health care dollars.^5^

**Figure 1:**
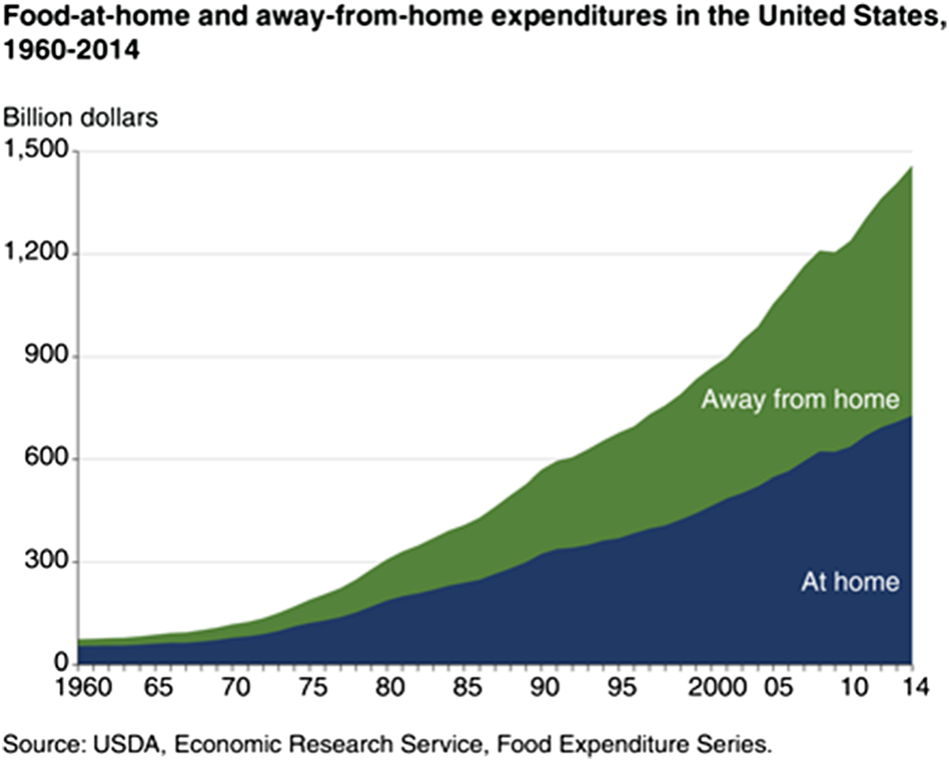
Food‐at‐home and food‐away‐from‐home expenditures in the US 1960‐2014

While making healthy dietary decisions when consuming food‐at‐home (FAH) continues to be a challenge in the American diet, increased consumption of food‐away‐from‐home (FAFH) is the more contemporary challenge. In 1980, expenditures on FAFH accounted 39% of all food dollars. Currently, as shown in Figure 1, approximately half of food expenditures are devoted to FAFH.^6^ While an increase in food expenditure away from home does not necessarily lead to a decrease in healthy dietary decisions, it is likely correlated with an increase in calorie consumption due to the larger portion sizes at restaurants.^7^ Consumption of FAFH, particularly fast food, is positively associated with increases in weight gain^8^, ^9^, ^10^, ^11^ and may lead to lower amounts of vegetable consumption, which contributes to poor diet quality.^7^ However, FAFH, particularly restaurant food, is not always positively associated with weight gain.^8^, ^12^ Recent research concluded that consumption of fast food and restaurant food is essentially equivalent in some nutritional quantities (ie, total calories, total fat, and saturated fat); and, in other nutritional quantities, restaurant food outperformed fast food (ie, lower intake of sugar and higher intake of certain vitamins and minerals); and, in other quantities, fast food outperformed restaurant food (ie, lower intake of cholesterol and sodium).^13^, ^14^, ^15^

The objective of this paper is to provide a narrative review of the history of nutrition labeling and effects of the respective nutrition information on food choice. Previous research has reviewed the literature for nutrition labels on packaged food,^16^, ^17^, ^18^ front‐of‐package (FOP) nutrition labeling,^19^, ^20^ and nutrition labeling on menus.^21^, ^22^, ^23^ The present review adds to this literature by examining research for nutrition labels for both FAH and FAFH. Additionally, this review adds to the literature by discussing the changes to the nutrition facts label (NFL) and recent findings from eye tracking studies.

## 2 BACKGROUND ON FAH NUTRITION INFORMATION

### 2.1 Nutrition facts label

In the late 1960s, the proliferation of processed food motivated changes to nutrition labeling, which, at the time, was voluntary.^24^ Another contribution to the increased desire for nutrition information was the improved understanding of the relationship between diet and obesity. The increased understanding of the link between obesity and disease (eg, heart disease and cancer) and the difficulty of assessing the healthfulness of food items spurred the demand for legislative efforts on nutrition labels (processed and manufactured food will be referred to as packaged foods henceforth).^25^, ^26^ The NFL was established by the Nutrition Labeling and Education Act of 1990 (NLEA) and designed by the Food and Drug Administration (FDA) to communicate the nutrient profile of packaged foods and, ideally, assist consumers in making healthy dietary decisions when consuming FAH.^27^ Despite mandatory policies for packaged foods, nutrition labeling continues to be “voluntary” for raw food. The nutrition information for raw food is to be displayed by labels affixed to the food or to external materials in close proximity to the food items, such as shelf labels, signs, posters, brochures, notebooks, or leaflets.^28^

**Figure 2:**
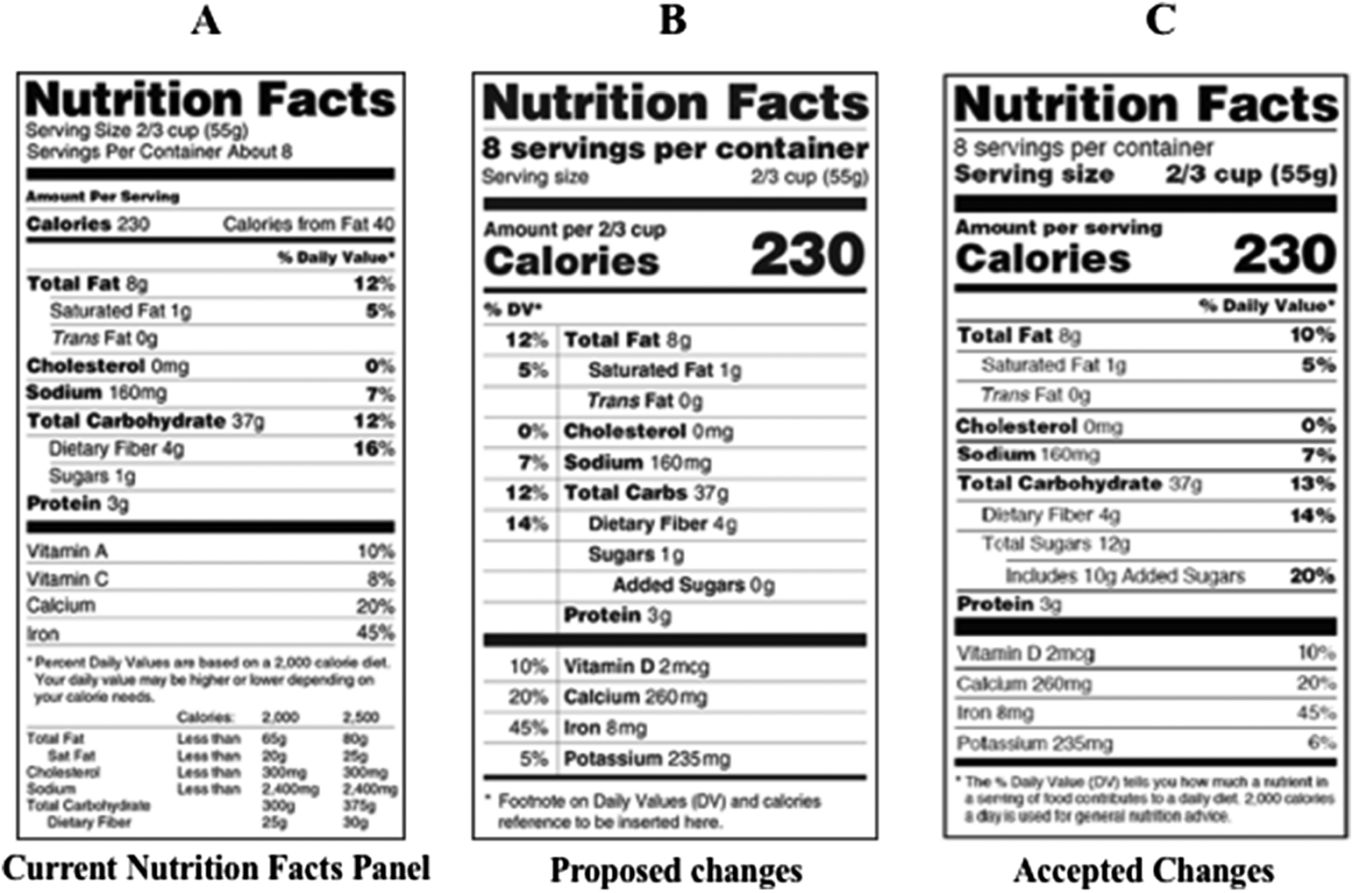
Current Nutrition Facts panel versus updated Nutrition Facts Labels. Major changes to the Nutrition Facts Label include increased font size and bolding of “Calories,” and “Servings Size.” Serving sizes have also been increased to reflect more realistic portion sizes. Additionally, “Added Sugars” is now teased out of “Total Sugars” and, now, there is a recommended daily intake for added sugar. “Calories from Fat” has been removed to indicate that not all fat should be avoided

The previous NFL, which has been present on most food products since 1994, requires information be provided for serving size, servings per container, calories per serving, calories coming from fat, amounts of macronutrients (ie, carbohydrates, fat, and protein), cholesterol, and sodium. Additionally, the previous NFL uses a base 2,000‐calorie diet to provide the percentage of recommended daily value per serving for total fat, saturated fat, cholesterol, sodium, total carbohydrates, dietary fiber, and micronutrients (ie, calcium, iron, vitamin A, and vitamin C).^29^ A depiction of the previous NFL is shown in Figure 2A.

The process to begin the revisions to the NFL began in 2005 and 2007 at the Advanced Notices of Proposed Rulemaking.^30^ Early revisions considered were removing “calories from fat,” recalculation of the “% Daily Values,” recommended intake values, and updating the “serving sizes” on packages.^30^ Prior to the revisions to the label, the only changes made to the NFL was the addition of “trans‐fat” content in 2006 due to evidence of trans‐fat intake increasing adverse effects in cardio‐metabolic health.^31^

In early 2014, the FDA suggested revisions of the previous NFL to ensure that consumers have access to nutrition information that reflect new scientific information about the linkages between diet and chronic diseases. A first draft of the updated NFL (Figure 2B) was proposed and public comments were elicited for the proposed changes.^32^ Some proposed changes were minor and simply made current information more pronounced to better highlight this information. To increase consumers’ attention to important information, the proposed NFL increased the prominence of “calories,” “servings per container,” and the numerical values of “calories” and “servings per container.” In another attempt to make important information more accessible, the percent daily value column was relocated from the right side of the label to the left side. Other changes suggested by the proposed NFL were more significant, for example, “calories from fat” was removed in an attempt to communicate that the type of fat consumed affects risks of chronic diseases relative to overall total fat intake. The proposed label also distinguished between natural sugar and added sugar by requiring the display of “added sugars.” Due to contemporary data about micronutrient deficiencies and the association of health‐related conditions, the requirements for the micronutrients displayed and certain daily recommend values were updated. Vitamin D and potassium, micronutrients that are underconsumed, replaced vitamins A and C on the new label. Finally, the proposed NFL suggested updating serving sizes to amounts that are more likely to be consumed. This includes the addition of another column that communicated all the nutrition information for the entire package if a food could reasonably be consumed on one occasion.^32^

In late May 2016, the FDA announced the finalized revisions to the NFL (Figure 2C).^33^ Ultimately, the changes made to the NFL were based on contemporary nutrition research, actual dietary advices from nutritional expert groups, and public opinion on the previous proposed changes. Elements from the 2014 proposed changes that were maintained in the finalized revision to the NFL included increased prominence of “calories,” and “servings per container,” and the numerical values of “calories” and “servings per container.” The change to serving sizes based on amounts of food and beverages that are actually consumed was maintained as well. The main differences between the 2014 proposed changes and the finalized updates to the NFL included the “serving size” line is now bolded, whereas “servings per container” is not. The percent daily value column remained on the right side of the label because research demonstrated the negative effects of moving the percent daily values to the left side.^34^ While the essence of “added sugars” was maintained in the final revision, the meaning was communicated more precisely and percent daily value was added. Laquatra et al^35^ concluded that the addition of “added sugars” confused the consumers and, thus, recommended adding more clarification. Therefore, the updated NFL displays “total sugars” with the addition of “includes (x) g added sugars.” Food manufacturers had until late July 2018 to comply with the final requirements and provide the updated NFL.^35^

### 2.2 FOP label

In late 2009, Dr. Margaret A. Hamburg, the then commissioner of FDA, wrote an open letter to the food industry highlighting the importance of providing nutrition information that consumers could rely on.^36^ She also expressed concerns about unauthorized health and nutrient content claims in addition to the unauthorized use of terms such as “healthy.”^36^ The letter also discussed making nutrition labeling a priority for the FDA, which was also supported by the then First Lady Michelle Obama *Let's Move!* initiative. Both the FDA and the first lady asked the industry to develop an FOP labeling system that would assist consumers in making more informed decisions.^36^

**Figure 3:**
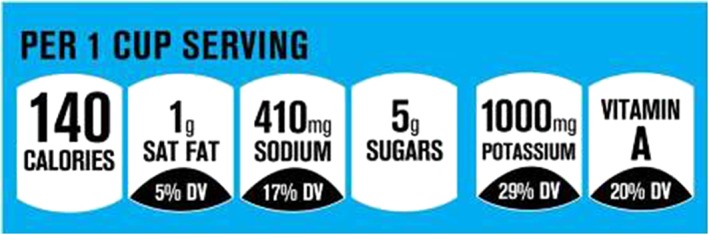
Facts Up Front front‐of‐pack nutrition labeling system

**Figure 4:**
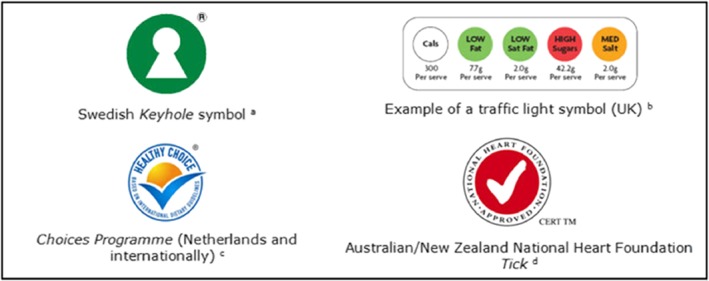
Examples of front‐of‐package labels worldwide

In response, the Grocery Manufacturers Association and the Food Marketing Institute developed the voluntary front‐of‐pack nutrition labeling system *Facts Up Front* (formerly called Nutrition Keys).^37^ *Facts Up Front* summarizes important nutrition information in an easy‐to‐use label on the front of food and beverages packages. As shown in Figure 3, the four basic icons are for calories, saturated fat, sodium, and sugars, which represent the key daily nutrients. As an option, labels can also include “nutrients to encourage” (ie, potassium, fiber, protein, vitamin A, vitamin C, vitamin D, calcium, and iron) and some nutrients were allowed to be placed on FOP if the product contained more than 10 percent of daily value per serving.^38^ *Facts Up Front* is not the only FOP nutrition labeling system. There are more than a dozen FOP labeling systems that have been developed and tested in various countries (Figure 4). For example, Sweden created the *Keyhole* program, Britain used the traffic light system based on a nutrient‐profiling approach designed by Oxford University, The Netherlands used the *Choices* program, and Australia created a National Heart Foundation label Tick for heart‐healthy approved foods.^39^

Bix et al^40^ demonstrated the impact of FOP labeling on the act of buying and developed a system to classify FOP labeling, ie, directive, nondirective, and semidirective. These categories correspond to the degree in which they provide guidance about the overall healthfulness of the product. Nondirective labels, which refer to the previous NFL and the Nutrition Keys system, provide a listing of nutrients that allow the consumer to correctly interpret the nutritional value of the food item. When nondirective labels are overlaid with symbols or other qualitative assessments (eg, color), these labels are described as semidirective. The color‐coded system provides an interpretation of the healthfulness of each component in reference to predetermined reference amounts. Finally, simple icons, like the Swedish Keyhole or the *Great for you* used by Walmart, are examples of directive labels that affirm food is nutritious.^40^

## 3 EFFECT OF FAH NUTRITION INFORMATION ON BEHAVIOR AND CHOICE

### 3.1 Previous NFL

Since the implementation of the 1990 Nutrition Labeling and Education Act, numerous studies have examined the effects of the previous NFL on consumer behavior. Many studies identified the effects of sociodemographic determinants on nutrition label use and have highlighted the necessity of nutrition education to fight obesity and other diet‐related health issues, which are correlated with individuals who use nutrition labels. These studies were conducted to examine factors that lead to NFL usage, and which factors are insignificant toward NFL usage. The earliest studies from the 1990s focused on age. In several studies, age did not have a significant effect on nutrition label use, as no specific age range was more likely to regularly use nutrition labels.^41^, ^42^, ^43^, ^44^, ^45^, ^46^ Others have demonstrated that age has a positive effect on label use, as younger consumers are more likely to understand the labels and perform label‐related tasks easier.^47^, ^48^, ^49^ Many studies have determined that women are more likely to use nutrition labels than men.^41^, ^45^, ^46^, ^47^, ^49^, ^50^ Finally, education and nutrition knowledge are associated with label use and understanding. Consumers, who have higher levels of education and nutrition knowledge, are typically able to comprehend label information and compare foods using labels easier than others.^41^, ^45^, ^47^, ^50^, ^51^, ^52^, ^53^, ^54^, ^55^

Some research has focused solely on the behavior of college students. The rational being is that some students adapt dietary habits in college, which remain throughout adulthood. However, it should be noted that convenience samples, like college students, may introduce bias that can be resolved with the inclusion of young adults not in college. Todd and Variyam^52^ reported that there has been a decline in consumer use of nutrition labels when making food purchases over the last decade, especially among young adults under 30. Even when college students agreed that the previous NFL was useful^47^ and easy to read,^41^ most of them do not use the label and doubt the accuracy and truthfulness of food labels. However, some research concluded that the NFL is useful when comparing two products^41^, ^48^ or when purchasing a food item for the first time.^41^, ^47^

Consumer use has decreased for most of the NFL components, such as calories, fats, cholesterol, and sodium. Despite these decreases, the use of fiber information has increased, whereas the use of sugars remained constant. Possible reasons behind the decline in consumer use are the difficulty of calculating the correct amount of nutrient intake, the increase in consumption of FAFH, the increase of the availability of nutrition information for FAH and FAFH online, and the fact that consumers are paying more attention to other information, such as country of origin, organic certification, or welfare issues.^52^

Many studies have critiqued the effectiveness of the NFL. In general, studies have demonstrated that consumers have a limited focus on the pervious NFL and do not examine every component of the label in detail.^56^, ^57^ Goldberg and Probart^57^ determined that the information located at the top or bottom of the label received more visual attention than the information located near the center of the label. They concluded that consumers who do not use nutrition labels often were more likely to view the information on the top of the label, and high frequency users focused on the information located near the center of the label, especially nutrient information.^57^ In a more contemporary study, Graham and Jeffery also determined that consumers focus on the top of the NFL, however, significantly less focus on the bottom lines of the label.^56^ Several studies have concluded that consumers are more interested in specific nutrition information, such as total fat and calories, and relatively less interested in minerals, trans fat, sugar, and dietary fibers.^41^, ^47^, ^56^ Finally, some studies revealed that consumers do not understand some specific vocabulary present on the label. Dallas et al^58^ explained that consumers generally misinterpreted the serving size information and believed that it defines the quantity of food that they can or should consume, whereas, in actuality, it refers to how much typical consumers eat, even if it is not the healthiest. Similarly, there are misinterpretations of specific terms such as “serving size,” “good source,” and “reduced calorie,” especially among college students.^41^

Most research has concluded that the NFL is generally misunderstood and misused. When analyzing the label of a single food item, studies report that the NFL is an inadequate tool, especially to plan diets or to follow dietary recommendations.^48^, ^51^ In particular, nutrition labeling can be difficult for consumers to assess when mathematical calculations are necessary, as any quantitative task may represents a barrier to information.^48^, ^52^ Rothman et al^51^ demonstrated that patients struggled to understand the previous NFL, indifferent of their literacy, and numerical skills. Misunderstanding the labels may lead to a false estimation of the quantity of nutrients consumed, and in particular, it may possibly contribute to a misinterpretation of the daily value percentages.^51^

The weaknesses of the NFL formulation led to research to suggest potential improvements to the NFL. An earlier change implemented in 2006 was the inclusion of trans‐fat information. Wang et al^59^ analyzed the effect of this change on demand for margarine and spreads and concluded that the change was successful in its early years of implementation but less effective in the long run. Later suggestions addressed issues such as diet and chronic diseases. Graham and Jeffery suggested to relocate important nutrients, like sugar, to a higher position on the label since consumers are more likely to read the top lines of the NFL.^56^ Rothman et al suggested highlighting “serving size” and “servings per container,” as well as providing nutrition information for the entire container of small food products to reflect actual consumption behavior.^40^ Finally, Todd and Variyam suggested creating awareness campaigns specifically targeting young adults to increase the use of nutrition labels.^52^

### 3.2 Revised NFL

After the FDA announced potential revisions to the previous NFL, some studies were conducted to test the effects of all of the proposed changes.^34^, ^60^ Some studies focused on specific proposed changes, such as the increase of serving sizes^58^, ^61^ or the inclusion of “added sugars.”^34^

Xie et al^60^ used eye tracking to examine consumer attention to the proposed changes to the NFL and concluded that the proposed NFL changes significantly increased consumer's attention; however, the degree of attention difference varied by product. They concluded that consumers spent more time viewing labels for relatively healthy food (ie, Healthy Choice Frozen Meal) because the negative nutrition information for relatively unhealthy food was easier to notice (ie, chips).^60^ Furthermore, the proposed NFL increased attention for consumers who previously had low involvement and were less familiar with a specific food product used in the experiment.^60^

Another eye‐tracking study, by Graham and Roberto,^34^ examined the effects of the proposed changes on visual attention and food choice of young adults and concluded that, when compared to the original NFL, the proposed NFL changes did not increase visual attention. Food choices were not significantly different between the label groups either. Additionally, the increased font size of “calories” and “serving size” did not significantly increase visual attention, which is contrary to the findings of Xie et al.^60^ The dissimilarity in results between the two studies may be due to differences in sample characteristics (ie, age). However, the “added sugars” line garnered more visual attention in young adults. Finally, Graham and Roberto reported that the proposed change of moving “%DV” from the right side to the left side of the NFL garnered less attention for this information.^34^

Several studies focused on the proposed change to update serving sizes to better reflect actual consumption of a food product. Interestingly, the updated “serving sizes” has been found to both increase^58^ and decrease^61^ consumption. Dallas et al^58^ suggested to add a serving size definition to the updated NFL because consumers use this information as a reference for consumption levels. Therefore, the increase of serving sizes could result in serving larger portions for themselves or for others.^58^, ^62^ In contrast, Hydock et al^61^ demonstrated that larger serving sizes could lead consumers to perceive some products as less healthy and, therefore, reduce the consumption of high‐calorie foods. Dallas et al^58^ determined that perception of serving sizes, as a reference point, resulted in participants overconsuming foods, whereas Hydock et al^61^ reported that participants had greater attention to nutrition information and, therefore, were sensitive to the increases in negative nutrient information.^61^ However, Hydock et al did note that the smaller serving sizes on the previous NFLs may reduce consumers’ guilt and, therefore, increase their own consumption.^61^

Laquatra et al^35^ focused on the inclusion of the “Added Sugars” line in the proposed NFL. Even though the addition of the “Added Sugars” line increases visual attention, consumers seem to misunderstand the actual meaning of “Added Sugars.” They note that, if the primary motivation of the FDA was to clarify the nutrition information and make it easier to understand for the consumers, it seems that this proposed change has led to more misinterpretations than the previous NFL. However, they determined that the combination of “Total Sugars” and “Added Sugars,” rather than “Sugars” and “Added Sugars,” helped clarify the meaning of the “Added Sugars.”^35^

A more recent eye‐tracking study on the proposed NFL found mixed results.^63^ The study used a variety of food products to test for the effect of product differentiation on the use of the previous and revised NFL. The authors concluded that the healthiness of the product determined the amount of visual attention paid to the proposed NFL. More visual attention was given to the healthier products in the experiment (salads, yogurt, and healthy frozen meal), whereas less healthy products (cereal, cookies, and potato chips) received less visual attention. Salad, which was the healthiest food option, and chips, the least healthy option, both received less visual attention than the other products. The authors posit this is due to “perceived ambiguity,” because the healthiness of the other food options was not as obvious as salad or chips. Due to the mixed results, the authors concluded that versions of the NFL should vary by food.^63^

### 3.3 FOP label

Discussions and possible implementations of FOP labeling are vast and, as with any nutrition‐labeling program, various FOP labeling has heterogeneous effects. The FOP labeling is very prominent in Europe, Australia, and New Zealand. In a study in The Netherlands on FOP labels, eye‐tracking and self‐reported measures have shown that, when consumers are looking for a specific nutrient with a specific health goal in mind, they will compare different products for the specific nutrient rather than closely examining the label of one product.^64^ Moreover, nutrition labels, even FOP label, are not the most viewed portion of a package and that they are viewed even less under a time constraint.^64^

The FOP labels that focus on colors, such as Traffic Light and the 5‐Color Nutrition Label, have received substantial attention in previous research.^65^, ^66^, ^67^, ^68^, ^69^, ^70^, ^71^ In a US study, it was reported that color‐coded labels were more effective than the NFL in attracting consumer attention regardless of the healthiness of the food.^65^ Balcombe et al^66^ concluded that UK consumers understood the labels and were interested in less consumption of a nutrient with the “red” label. Traffic Light labels are also effective under time constraint,^67^ and eye‐tracking results have confirmed that less time is needed to process Traffic Light labels than Guideline Daily Amount labels.^68^ Crosetto et al^67^ asked participants to plan a daily menu using either Guideline Daily Amounts or Traffic Lights, and, while Guideline Daily Amounts are more informative, Traffic Lights were as effective when time was constraint. Moreover, eye‐tracking results have confirmed that less time is needed to process the Traffic Light labels.^68^ Ducrot et al^71^ concluded that color‐coded labels were the most effective for helping consumers rank food items based on healthiness. When testing for the nutritional quality of food choice, the 5‐Color Nutrition Label outperformed the Guideline Daily Amounts, Traffic Light, and Green Tick Label.^69^ Of the labels studied, Guideline Daily Amounts was the least effective at improving nutritional quality.^69^ Julia et al^70^ confirmed the success of the 5‐Color Nutrition Label in its ability for consumers in the French market to differentiate the nutritional quality of breakfast cereals.

Other studies question the usefulness of color‐coded labels. Bialkova and Van Triijp^72^ concluded that monochrome labels were more effective than polychrome coloring. However, using purchasing data on yogurt products and ready meals from a major retailer in the UK, Boztug et al^73^ determined that monochrome labels contributed to healthier choices only when the data were aggregated, but purchasing behavior did not change when disaggregating the categories. Helfer and Shultz favored simpler FOP labeling schemes as opposed to Traffic Lights or Guideline Daily Amounts and concluded that Traffic Lights only contribute to moderate increases in more nutritious food choice.^74^

Additional FOP labeling systems include star‐ratings, like the Guiding Stars that are developed by the Hannaford supermarkets, in which a 0‐3 rating scale is used as recommended by the National Academy of Medicine (formerly known as the Institute of Medicine).^75^, ^76^ This system rates food based on how many key nutrients (fats, sugars, and salt) are over recommended limits.^77^, ^78^ Some research determined that stars make it easier to understand the healthiness of a product,^77^ whereas other research concluded that there was confusion in the healthiness of products using this system.^78^ The Guiding Stars used stars that ranged from zero to three to communicate the healthfulness of a product; however, it has been argued that having zero stars removes a reference point that can be used to evaluate the differences in attributes.^79^ Graham and Mohr's first experiment used a 0‐3 star ranking and concluded that food with zero stars were considered healthier than food with one star and equal in healthiness to food of two stars.^78^ In the second experiment, using a 1‐4 star ranking, the new reference point is allowed for a clearer understanding of the label and thus healthier choices.^78^ Lundeburg et al^80^ saw conflicting results as to Graham and Mohr^78^; they conducted an experiment on college students where they asked them to view products and rate them on healthiness. They concluded that the star labeling system was most efficient at participants making healthy choices, as it outperformed the Traffic Light labels. The Guiding Stars were successful at deterring consumers away from food that was deemed “very poor” in nutrition quality.^81^ Rahkovsky et al^82^ tested for the effectiveness of the Guiding Stars Program on ready‐to‐eat cereals and concluded that healthier cereals were purchased if price was held constant. Furthermore, Sutherland et al also showed that the Guiding Star Program was effective at changing consumer choice over the course of multiple years.^83^

Australia has a similar labeling design to the Guiding Stars, known as the Health Star Rating. Neal at al measured the effectiveness of the Health Star Rating label against other labels, such as the Traffic Lights, and, while the Health Star Rating label was the most preferred by consumers, it did not result in healthier food choices.^84^

Bix et al^40^ concluded that FOP labels are effective, as they increase attention to nutrition information. In particular, the color‐coded system increases the consumers’ attention to nutrition information. However, they also concluded that FOP labeling can be used as a short‐cut under certain situations, and it decreases consumers’ attention to the information provided by the NFL on the back of the package. Therefore, Bix et al^40^ strongly suggested that the most important information should appear in the FOP label. On the contrary, Turner et al suggested that FOP labels are not short‐cuts when consumers are explicitly interested in nutrition information and concluded that consumers with motivation to buy healthful food spend more time looking at all available nutrition information, in comparison to consumers who purchase based on taste.^85^

In a recent study, Graham et al^86^ quantified NFL and FOP label viewing using eye‐tracking technology and examined differences between participants who viewed NFL vs FOP. The results indicated that NFL were less likely to be viewed than FOP labels during a food‐selection task and the authors concluded that increased visual attention for FOP labels occurred because of signage that was present in the grocery store at the time that informed consumers about the purposes of FOP labels. According to this study, FOP labels are only relevant if an awareness campaign to educate consumers on the availability of this resource accompanies its usage.^86^

It will be more efficient to keep the Nutrition Keys system as noncompulsory and create awareness campaigns to educate American consumers, so they could use this label to make healthier food choices.^87^ Furthermore, effective FOP labels would facilitate the comparison between several similar products available on a supermarket. The effectiveness of any given system may vary with the population's nationality, culture, level of health literacy, and other socioeconomic status.^88^ Andrews et al^89^, ^90^ revealed the importance of giving an education to the American citizens to contribute to a deeper understanding of how nutrition icons work. In summary, the findings indicated that continued examination of FOP system is warranted to enhance the system.^87^, ^88^, ^89^, ^90^

## 4 BACKGROUND ON FAFH NUTRITION INFORMATION

Food sold at fast‐food and sit‐down restaurants was exempted from the Nutrition Labeling and Education Act of 1990.^27^ In December 2006, the Center for Science in Public Interest (CSPI) collaborated with the New York City's Department of Health and Mental Hygiene on the first menu labeling policy, requiring calories labeling on menus and menu boards of fast‐food and chain‐food restaurants.^32^ In September 2008, Arnold Schwarzenegger, then governor of California, passed the first state menu labeling legislation. The CSPI collaborated on the development of the bill. Henceforth, CSPI has helped and continues to advocate for menu labeling policies in more than 20 states, counties, and cities. As part of the Affordable Care Act, the US Congress adopted a national law for calorie labeling on menus, menu boards, and food on display at restaurants and other similar retail establishments that have at least 20 locations are doing business under the same name or offering similar food items to restaurants.^39^ In December 2014, the FDA finalized menu and vending labeling regulations. In May 2016, the menu labeling implementation guide was finalized and has been enforced since May 2017.^32^ However, many fast‐food and sit‐down restaurants displayed caloric information on menus prior to enforcement. Consequently, some studies have been conducted to determine if legislation requiring menu calories has a real impact on food choice for FAFH.^34^

## 5 EFFECT OF FAFH NUTRITION INFORMATION ON BEHAVIOR AND CHOICE

Cafeterias have provided a setting for several studies^91^, ^92^, ^93^ Research in cafeterias has shown an impact on both intentions to select food^92^ and actual choice.^91^, ^94^ Thorndike et al^93^ assessed the effectiveness of color‐coded labeling in a cafeteria and determined that sales of unhealthy items (coded red) decreased and sales of healthy items (coded green) increased significantly. The largest decrease in unhealthy items was noticed in the beverage category. Additionally, the impact on choice was more noticeable when combining color coding and positioning items in a more convenient location.^93^

Several studies focused on different dimensions of fast‐food menu labeling.^94^, ^95^, ^96^ Self‐reported attention to calorie labeling is associated with total calories purchased^94^ and may have contributed to a 1.5% reduction in body mass index (BMI) and a 12% reduction in persons with obesity.^95^ Restrepo^95^ used state files from the 2004‐2012 Behavioral Risk Factor Surveillance System to compare health measurements in counties that implemented labeling laws, and counties that did not. The presence of menu labeling is correlated with a 1.5% reduction in BMI, and a lowered risk of obesity (12%), when compared to time periods prior to implication. Calorie labeling in New York was also associated with body weight reductions, especially in lower income minority groups.^95^ An eye‐tracking study examined the effect of three label formats on attention, ie, numeric, color‐coded, and physical activity‐based formats.^96^ The physical activity‐based labeling, which put caloric information into how much physical activity it would take to burn the calories, was the most preferred and effective type of label. The physical activity labels attracted the most visual attention, and the customers made healthier food choices when they were present.^96^

While the previous studies are informative, a control group was not included to determine if changes intentions or choice occurred randomly. Ellison et al^97^ examined food choice in a sit‐down restaurant where patrons were randomized to a menu‐labeling treatment. Two label treatments provided calorie information (one with the number of calories and one that used symbols to communicate calorie content), and a control menu that did not provide any information about calorie content. While both label treatments influenced food choice, effectiveness of a menu label varied based on the level of knowledge consumers had about nutrition. The effectiveness of the labels is determined by calories purchased. The numeric representation of calories reduced caloric intake for consumers with relatively less knowledge about nutrition and the menu with the symbolic calorie label was more effective in reducing calorie intake for more knowledgeable consumers. Consumers of lower health consciousness were affected more by the implementation of the nutrition labels than those of high health awareness.^97^

Other studies have examined the impact of menu labeling by using similar cities without menu labeling as a control.^98^, ^99^, ^100^ Including a control location allows for a difference‐in‐difference analysis. Finkelstein et al^98^ examined the impact of menu labeling in King County, Washington. The analysis examined transactions before and after menu labeling at seven locations in King County and seven control locations. Results indicated that calories per transaction did not vary between King Country and the control locations after calories were displayed on menus. Elbel et al^99^ examined the impact of menu labeling in Philadelphia by eliciting self‐reported use of calorie information and determined calories purchased from fast food receipts from consumers leaving restaurants. Baltimore was used as a control because it was a similar city that did not have menu labeling. Significantly, more consumers in Philadelphia self‐reported noticing calories on menus, which is not surprising, given that there was no menu labeling in Baltimore. However, the difference in the number of fast food visits or calories purchased was not significant between the two cities. These results do not provide evidence that mandatory menu labeling positively influenced food purchasing behavior. Elbel et al^100^ used Newark, New Jersey as a control to examine the impact of New York City's labeling mandate. The results indicated that 27.7% of those who saw calorie labeling in New York said that the information influenced their choices; however, there was no noticeable change in calories purchased.^100^

## 6 CONCLUSION

The American diet is increasingly energy rich but nutrient poor. This is indicative from the high prevalence of obese persons in the United States, and the 2015 Dietary Guidelines Advisory Committee report declares that Americans underconsume calcium, fiber, iron, potassium, and Vitamin D and overconsume saturated fat and sodium.

Nutrition labeling was established because it is impossible for consumers to determine the nutritional content of packed and prepared food even after consumption. Continuing research provides a better understanding of how to help consumers develop a diverse diet. The proposed changes to the NFL, as well as the inclusion of FOP labeling and menu labeling for FAFH, represent the evolution of knowledge about attention to information and another step in the process to better inform consumers.

Nutrition information is often put forth as a way to change food choice; however, as research has shown, simply providing information is not that effective.^16^, ^17^, ^18^, ^19^, ^20^, ^21^, ^22^, ^23^ A problem may be that many consumers use the nutrition labels to avoid certain nutrients that often is accompanied with conflicting information (eg, fat and sodium). Information to develop a diversified diet may be more effective if it is targeted to specific audiences.

More research is needed to understand the effectiveness of consistent labeling for FAH and FAFH. It is impossible to list as many details on menu labeling that can be included on products in the grocery store, and understanding nutrition labeling depends on the ability to understand quantitative information. However, labeling cues, such as color coding, could be consistently displayed for consumers when shopping for FAH and FAFH. Of course, any nutrition labeling is dependent on the accuracy of claims, which may be more problematic for FAFH.^101^ Nevertheless, there may be innovative ways to display nutrition information, which may make it more accessible to most consumers. For example, providing per‐meal recommendations with nutrition information would provide a reference point and allow consumers to deliberate tradeoffs between meals. 102 A nutrient‐to‐energy ratio, or some type of index, may help consumers better understand nutrient density within and across food groups and FAH versus FAFH.

Another potential consideration is to improve awareness campaigns to increase knowledge of nutrition and improve understanding of labels. As noted in the work of Graham et al,^87^ it is more efficient to keep FOP schemes to effectively compare food items. The same reasoning could be applied to the NFL. An effective awareness campaign paired with clear formulation of the NFL can allow for easy comparisons of healthfulness in food items at the supermarket. Much like FOP labeling, the effectiveness of the NFL is contingent on the nationality, culture, level of health literacy, and socioeconomic status.^88^ The current lack of awareness and understanding of the NFL indicates its inability to effectively improve diet quality in the United States.

## FUNDING

None to report.

## DISCLOSURE

The authors declared no conflicts of interest.
